# Lectotypification of *Chamaecyparishodginsii* of the Cupressaceae

**DOI:** 10.3897/phytokeys.185.75796

**Published:** 2021-11-18

**Authors:** Zhi Yang, Yong Yang, Keith Rushforth

**Affiliations:** 1 College of Biology and the Environment, Nanjing Forestry University, 159 Longpan Rd., Nanjing 210037, China Nanjing Forestry University Nanjing China; 2 The Shippen, Ashill, Cullompton, Devon, EN15 3NL, UK The Shippen, Ashill Devon United Kingdom

**Keywords:** *Chamaecyparishodginsii*, lectotypification, nomenclature, taxonomy

## Abstract

Recent phylogenetic studies have suggested that the monotypic *Fokienia* A.Henry & H.H.Thomas is nested within *Chamaecyparis* Spach, which is in agreement with separate morphological studies. Here the authors confirm a previous taxonomic treatment that incorporated *Fokieniahodginsii* (Dunn) A.Henry & H.H.Thomas into *Chamaecyparis* based on the monophyly requirement of taxonomy, i.e. *Chamaecyparishodginsii* (Dunn) Rushforth. In addition, the type collection of the basionym *Cupressushodginsii* Dunn was found to contain three sheets of specimens, one in K including a vegetative branch (K000088294) and a separate ovulate cone (K001090486), a second one in A (A00022477), and a third one in IBSC (IBSC0016081). All three specimens are marked with Hongkong Herbarium No. 3505, but only the two specimens in K and IBSC possess similar handwriting of “*Cupressushodginsii* Dunn”. The two specimens should be considered as syntypes according to the Shenzhen Code. The specimen in K is better preserved but it is a mixture according to the collection label: cones from Foochow (Fuzhou) and foliage from Yenping (Nanping). We lectotypified the name *Cupressushodginsii* with K000088294 because the specimen is well preserved and has enough characters for identification. Moreover, an ovulate cone (K001090486) is on the same sheet.

## Introduction

Dunn (1908) described a new conifer species from Fujian of China, *Cupressushodginsii* Dunn. Henry and Thomas (1911) believed that this species is different from *Cupressus* L. in the cone scale having only two seeds with two very unequal lateral wings, and should be classified into a separate genus, establishing *Fokienia* A.Henry & H.H.Thomas (generic name is after the provincial name Fujian where the type was collected) and transferred the species into this monotypic genus, i.e. *F.hodginsii* (Dunn) A.Henry & H.H.Thomas. Subsequently this monotypic genus was widely accepted and well known in botanical literature. Both *Flora Reipublicae Popularis Sinicae* (vol. 7, Wang et al. 1978) and *Flora of China* (vol. 4, Fu et al. 1999) treated it as a monotypic genus.

Since 2000, molecular systematic studies have consistently suggested that *Fokienia* is close to *Chamaecyparis* (Gadek et al. 2000; Yang et al. 2012; Qu et al. 2017; Stull et al. 2021). Based on a morphological study, Rushforth (2007) thought that the morphological variation of the genus *Fokienia* overlaps or falls within the range of *Chamaecyparis* including ovule number per cone scale, seed wing symmetry, and time duration for cone ripening, and thus reduced the genus to synonymy with *Chamaecyparis*, and made the combination *Chamaecyparishodginsii* (Dunn) Rushforth. This morphological taxonomy was supported by a few subsequent phylogenetic studies (Mao et al. 2010, 2012; Jagel and Dörken 2015). Mao et al. (2012) sampled all extant five species of *Chamaecyparis* and the only species of *Fokienia*, assembled a sequence data including chloroplast, mitochondrial, and nuclear ribosomal DNA, and reconstructed a robust phylogeny of Cupressaceae; they demonstrated that *Fokienia* is nested within *Chamaecyparis*, with *Fokieniahodginsii* as sister to a subclade of *Chamaecyparis* including *C.obtusa* and *C.lawsoniana*, which received high bootstrap supports. The taxonomic treatment of Rushforth is acceptable if the monophyly principle is applied, and *Chamaecyparishodginsii* should be the correct scientific name of the species.

*Chamaecyparishodginsii* is a flag species of conservation. Fu (1992) recorded *Fokieniahodginsii* (≡*Chamaecyparishodginsii*) in his China Plant Red Data Book: Rare and Endangered Plants; Qin et al. (2017) listed this species as threatened (VU). *Chamaecyparishodginsii* is widely but sporadically distributed in southern China, Laos, and Vietnam. It has become a vulnerable species for a number of reasons, e.g. habitat loss, over exploitation, many old trees died of diseases, and difficult population regeneration (Chen et al. 2018). The recently released List of National Key Protected Wild Plants of China (ver. 2021) follows the treatment of the List of National Key Protected Wild Plants of China (Batch 1, ver. 1999), and lists the protection status of *Fokieniahodginsii* (≡*Chamaecyparishodginsii*) as Grade II of protection.

**Figure 1.**
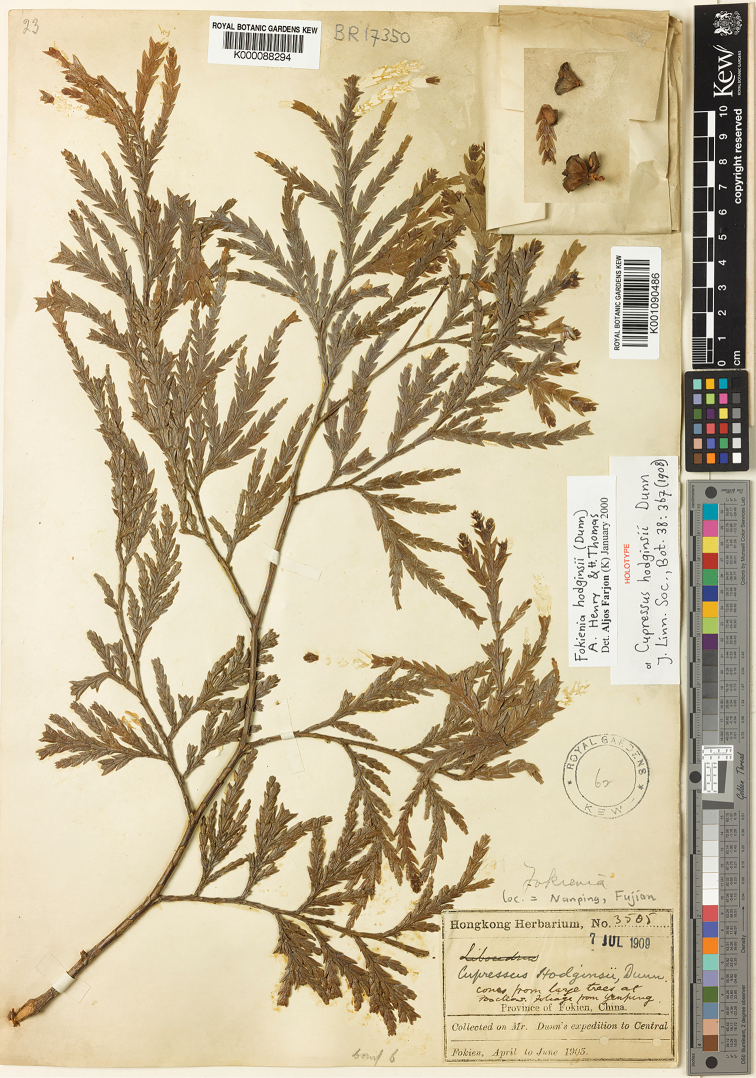
Lectotype of *Chamaecyparishodginsii*: Hongkong Herbarium No. 3505 (K000088294).

In the protologue of *Cupressushodginsii* Dunn, Dunn (1908) indicated a single collection, i.e. Hongkong Herbarium No. 3505, which should be considered as the type though he did not designate the herbarium where he deposited the type specimen. We found three duplicates of the collection: one is deposited in the Kew Herbarium (Royal Botanical Garden, Kew) (Fig. 1) and includes a vegetative branch (K000088294) and a separate ovulate cone (K001090486); the label says “cones from large trees at Foochow, foliage from Yenping, Province of Fokien, China”; a second is in the Herbarium of South China Botanical Garden, Chinese Academy of Sciences (IBSC0016081), and a third is in Harvard University Herbaria (A00022477). Farjon (2010) considered the specimen in K is the holotype, which is obviously a mistake. The two duplicates in K and IBSC should be considered as syntypes under Art. 9.6 and 40.2 Note 1 of the *Shenzhen Code* (Turland et al. 2018), both were studied by Dunn because the identification label is marked with his handwriting while the sheet in A should be considered as an isosyntype because it lacks any handwriting label. A lectotype should be designated under Art. 9.11 and 9.12. The specimen in Kew is better preserved, and should be considered as the lectotype. However, that specimen is a mixture according to the collection note that the vegetative branch was collected from “Yenping (Nanping)” and the ovulate cone was from “large trees at Foochow (Fuzhou)”. Here we designate the vegetative branch (K000088294) as the lectotype. This lectotype specimen clearly displays a few morphological characters of the species, i.e. dimorphic leaves 4–10 mm long, decussate, facial pairs closely appressed, lateral pairs boat-shaped and having two white depressed stomatal bands abaxially.

## Typification

### Chamaecyparis hodginsii

Taxon classificationPlantaePinalesCupressaceae

(Dunn) Rushforth, J. Biol. (Vietnam) 29(3): 38 (2007)

2AE6AE12-0417-5549-BED4-798580D12C80

Fig. 1

≡Cupressushodginsii Dunn, J. Linn. Soc., Bot. 38: 367 (1908); Fokieniahodginsii (Dunn) A.Henry & H.H.Thomas, Gard. Chron. ser. 3, 49: 67 (1911).

#### Type.

China. Fujian (福建, as “Fokien”): Nanping (南平, as “Yenping” in the protologue), April to June 1905, A.E.N. Hodgins, Hong Kong Herb. 3505 (lectotype: K000088294, designated here; isolectotypes: IBSC0016081, A00022477).

#### Distribution.

China: Chongqing, Fujian, Guangdong, Guangxi, Guizhou, Hunan, Jiangxi, Sichuan, Yunnan, Zhejiang; Laos; Vietnam.

## Supplementary Material

XML Treatment for Chamaecyparis hodginsii
